# Fourier-Domain OCT Imaging of the Ocular Surface and Tear Film Dynamics: A Review of the State of the Art and an Integrative Model of the Tear Behavior during the Inter-Blink Period and Visual Fixation

**DOI:** 10.3390/jcm9030668

**Published:** 2020-03-02

**Authors:** Pietro Emanuele Napoli, Matteo Nioi, Lorenzo Mangoni, Pietro Gentile, Mirco Braghiroli, Ernesto d’Aloja, Maurizio Fossarello

**Affiliations:** 1 Clinica Oculistica, San Giovanni di Dio Hospital, Azienda Ospedaliera Universitaria di Cagliari, 09124 Cagliari, Italy; lollomangoni92@gmail.com (L.M.); gentilepietro92@gmail.com (P.G.); maurizio.fossarello@gmail.com (M.F.); 2 Department of Surgical Sciences, Eye Clinic, University of Cagliari, 09124 Cagliari, Italy; 3 Department of Medical Sciences and Public Health, University of Cagliari, 09042 Cagliari, Italy; nioimatteo@gmail.com (M.N.); ernestodaloja@gmail.com (E.d.)

**Keywords:** tear film, ocular surface, tear film dynamics, visual fixation, optical coherence tomography

## Abstract

In the last few decades, the ocular surface and the tear film have been noninvasively investigated in vivo, in a three-dimensional, high resolution, and real-time mode, by optical coherence tomography (OCT). Recently, OCT technology has made great strides in improving the acquisition speed and image resolution, thus increasing its impact in daily clinical practice and in the research setting. All these results have been achieved because of a transition from traditional time-domain (TD) to Fourier-domain (FD) technology. FD-OCT devices include a spectrometer in the receiver that analyzes the spectrum of reflected light on the retina or ocular surface and transforms it into information about the depth of the structures according to the Fourier principle. In this review, we summarize and provide the state-of-the-art in FD-OCT imaging of the ocular surface system, addressing specific aspects such as tear film dynamics and epithelial changes under physiologic and pathologic conditions. A theory on the dynamic nature of the tear film has been developed to explain the variations within the individual compartments. Moreover, an integrative model of tear film behavior during the inter-blink period and visual fixation is proposed.

## 1. Introduction

The tear film (TF) represents the first barrier of the eye against environmental harmful agents, ranging from infections to pollution [1,2]. Clearly, the synergic cooperation of a large amount of structures is essential to achieve such a complex objective. In this sense, the TF and the contiguous epithelia of the ocular surface (cornea, conjunctiva, main and accessory lacrimal glands, and the meibomian glands) play a fundamental role.

Although the analysis of the ocular surface is an indispensable step in the evaluation of the health status of the eye, no traditional instrument has proven to be singularly useful for the contactless, in vivo, cross-sectional, high resolution and overall assessment of its various structures at different levels of depth [3,4].

Moreover, the reflex tearing represents the main problem for the majority of common diagnostic minimally-invasive or invasive tests, since they potentially alter the environment under evaluation [5]. Therefore, a non-invasive imaging approach that elaborates the reflected light from the structure being scanned can permit disclosure of the “true” state of the lacrimal system.

Optical coherence tomography (OCT) is a relatively recent and fast, infrared-light-based imaging modality for evaluating various ocular elements in real time. This interferometric method has been used to explore the anterior segment of the human eye since 1994, and it has gradually become a reliable diagnostic tool in clinical practice [6,7,8].

Recently, the introduction of spectral-domain (SD) OCT technology has led to a significant increase in speed acquisition and in imaging resolution at specific depths, as well as in the signal to noise ratio [9,10]. All these improvements are related to the Fourier-transformation from the acquired spectra from the object being scanned, which is a peculiar feature in all Fourier-domain (FD) OCT systems.

Although other interesting FD-OCT technologies also exist, such as swept-source (SS) imaging platforms [11], they offer limited advantages over SD-OCT in the study of TF. Indeed, SS-OCT can provide greater scanning depth but not better resolution [12].

The aim of the present review is to summarize and provide the state-of-the-art in FD-OCT imaging of the ocular surface (mainly with SD-OCT), addressing a number of aspects such as natural/artificial tear film dynamics and ocular surface changes in different situations. Moreover, an integrative model of tear film behavior during the inter-blink period and visual fixation is presented, wherein the core assumption is the dynamic nature of the tear film across the individual compartments of ocular surface.

## 2. Literature Review

We searched Web of Science, Pubmed, Ovid, Embase and Scopus databases for original articles published from 1991–2019, using the following combination of keywords: OCT “artificial tears”, OCT “tear film”, OCT “tear meniscus”, OCT “ocular surface”, OCT “tear film dynamics”, without any limitation (using the Bolean operator ‘AND’). The reference list of retrieved studies was manually screened for further studies. The searches were performed by two independent investigators (P.E.N, M.N).

After removing duplicates, all titles and abstract of all identified citations were individually screened. Full texts of papers deemed as potentially eligible were obtained and individually screened for eligibility.

From a total of 4360 papers found using these searches, we only selected 104 articles for our review. Specifically, we excluded studies that were not focused on the physiology of the lacrimal functional unit, and papers without new insights on the functions or structure of the tear film and ocular surface.

## 3. Technical Features of OCT Imaging

**Figure 1:**
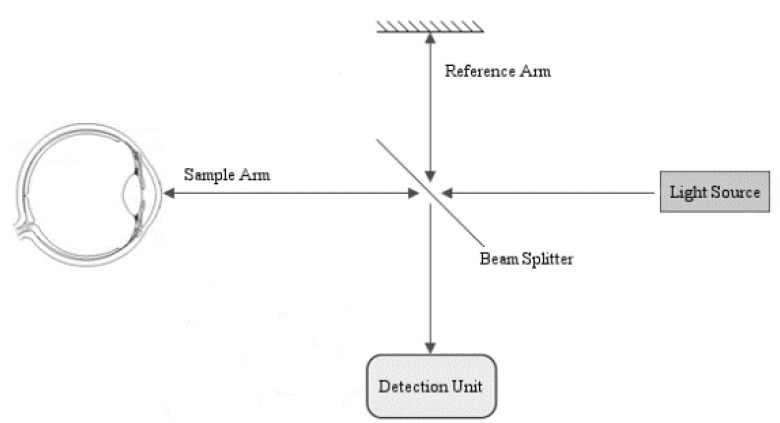
Schematic view of a Fourier-domain (FD) optical coherence tomography (OCT). Components include: reference mirror, sample (in our case, ocular structures), beam splitter, detection unit (a spectrometer or a point detector), and light source (a superluminescent diode or a swept-source laser) [13]. Reference mirror (the line at the top) with associated “reference arm”.

OCT is an imaging system, based on low-coherence interferometry, which measures the reflected light from the ocular structures being scanned (Figure 1) [13].

The light from narrow- or broad-bandwidth light sources is broken into two arms by a beam splitter: a reference arm (i.e., a mirror) that reflects the signal, and a sample arm where radiation is backscattered by the item of interest. As a result, the combination of the two signals determines an interference pattern only if the optical difference is less than a coherence length.

**Figure 2:**
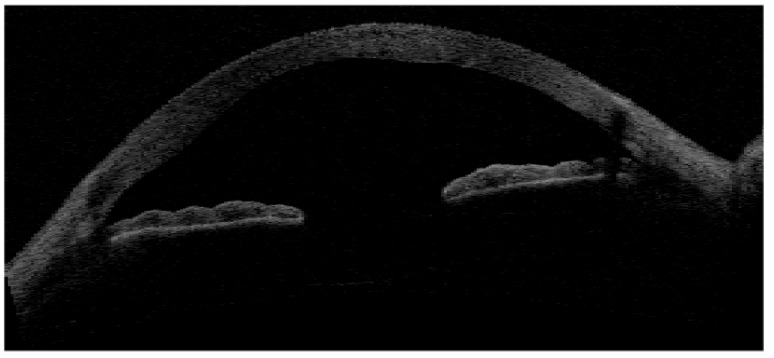
Time-domain (TD) optical coherence tomography (OCT) imaging of the anterior segment. Although the TD-OCT platform was commercialized as the first OCT system specifically conceived for anterior segment imaging (Visante OCT), it clearly provides an inadequate resolution to analyze in detail the fine changes in the tear dynamics and ocular surface epithelia. However, an interesting feature of TD systems may be represented by the wide field of view (scan depth and width) of the ocular surface profile and anterior segment structures.

SD technology is a type of FD-OCT system (with a superluminescent diode as light source), which gathers the light returning from the reference and the sample arm with all the spectral components detected simultaneously [14]. In detail, SD-OCT elaborates the reflected radiation by means of a complex and technologically advanced detection unit(spectrometer), which distributes various optical frequencies through a dispersive element on a detector band. This imaging system does not need any moving mirrors, since the depth scan is easily measured by a Fourier-transform from the detected spectra. In this way, higher acquisition rates (A-scans) compared to other technologies, such as time-domain OCT, are obtained (Figure 2).

Although SD-OCT was initially conceived for retina imaging [15,16], it was later adapted to visualize the anterior segment of the eye through the addition of a positive lens system [17]. For example, the Cirrus HD-OCT (Carl Zeiss Meditec, Dublin, CA, USA) adopted a 60-diopter aspheric lens for anterior segment.

**Figure 3:**
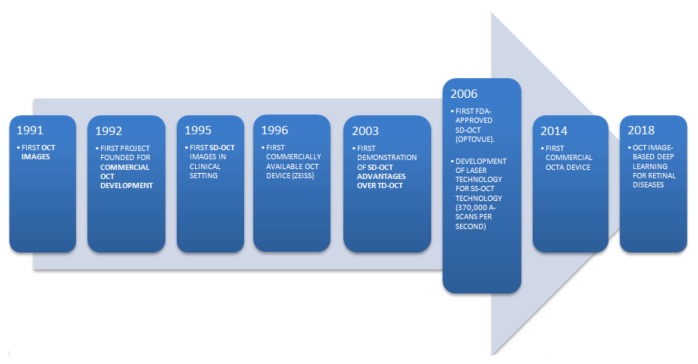
A timeline of optical coherence tomography (OCT) imaging. In 1991, David Huang was the first to demonstrate the applicability of low-coherence interferometry in the quantitative assessment of biological systems [13]. The transition from traditional time-domain (TD) to Fourier-domain (FD) technology, which includes spectral-domain (SD) and swept-source (SS) platforms, required clinical feasibility studies, entrepreneurship and business support in order to obtain clinical acceptance and further developments.

The SS and SD systems are very similar to each other, but the SS-OCT differs both in the light source (which emits a narrow bandwidth that is rapidly swept in frequency within the laser) and in the detection unit (i.e., a point detector), thus obtaining elevated scan speeds (A-scans per second (Figure 3) [11].

Currently, with regards to the tear film imaging, SD-OCT technology provides the best combination of axialand transverse resolution, which are two independent features of the device (unlike in conventional microscopy). For these reasons, the present review is mainly focused on SD-OCT results (as mentioned above).

## 4. Structure and Appearance of the Tear Film

The tear film-air interface is the primary and the most powerful refractive structure of the optical system of the eye (approximately 45 diopters). In the calculation of the ocular refractive power, the tear film has been in the past excluded due to its thinness and to the similarity between its refractive index and that of the cornea [18].

Human tears are known to be cyclically redistributed during blinking in three regions of the ocular surface, i.e., the preocular tear film, the menisci and the fornices.

Currently, a stable tear film over the epithelial cells is considered one of the hallmarks of overall ocular health, since it performs refractive, protective, moisturizing and nutritive functions [18,19].

The preocular TF adheres to the epithelia of cornea and conjunctiva through the mucinous structure of the glycocalyx in order to resist the gravitational forces and to balance the chemical and physical interactions of the system. An abnormality of this delicate system may result in a poor adhesion and, consequently, in an instability of the TF, with deterioration of vision.

As previously reported, a prolonged interruption of blinking may lead to a reduction of visual performance and optical quality [20], rapidly inducing higher-order wave front aberrations [21].

Overall, tears represent an interactive system including lipids, proteins, mucins and other compounds [22]. Although the TF is generally believed to be a dynamic structure composed of three layers (lipid, aqueous and mucin), this model is considered a “simplification of reality” by some authors [23,24]. Indeed, ocular surface mucins are distributed according to an increasing gradient of concentration from the aqueous layer towards the epithelium [25], thus suggesting that the aqueous and mucin layers should be considered as a single, “inseparable” layer of mucoaqueous gel [26].

**Figure 4:**
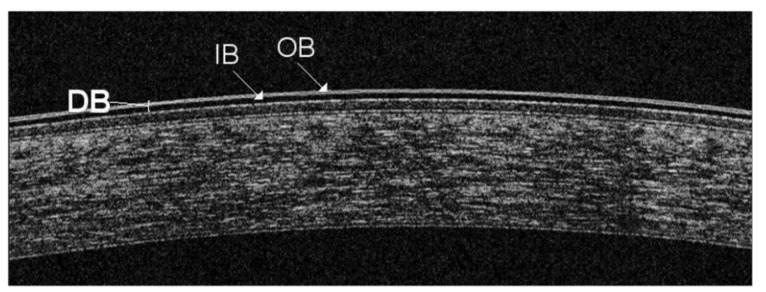
Enhanced imaging of the precorneal tear film (TF) by spectral domain (SD) optical coherence tomography (OCT). Although the axial resolution of SD-OCT systems (~ 5 µm) is close to the precorneal TF thickness in healthy subjects, TF can be increased in volume by means of an enhancer in order to be detected and monitored over time. Thus, a double-band structure (DB) of the precorneal TF, made by an inner (IB) and outer (OB) layer of opposite reflectivity can be visualized above the central cornea [27]. It is necessary to maintain a predetermined range of temperature (15 °C–25 °C) and humidity (30%–50%) in a dimly lit consulting room to standardize the OCT scanning protocol [4].

As a two-layered structure, the precorneal TF can be easily visualized and studied with OCT over time after an increase of its volume with an enhancer (Figure 4). This approach is useful to measure the adhesive forces between the corneal epithelium and an artificial TF, the sodium carboxymethylcellulose (NaCMC)-based compound [27]. In a vertically oriented ocular surface, the attractive forces that retain tear fluid in opposition to the gravitational forces (under the same environmental and bodily conditions, e.g., blinking) are clearly represented by the adhesive properties of the system. Therefore, we calculated the retention time of the adhesion marker above the central cornea, where only gravity dominates the tear film drainage [28]. This was possible because NaCMC is capable of binding to human corneal epithelial cells [29]. The OCT imaging results indicate that patients with dry eye or meibomian gland dysfunction (MGD) have a significant reduction of TF corneal adhesiveness [30].

From a theoretical point of view, the natural thickness of the TF, despite its dynamic nature, is generally believed to be too low for OCT resolution (~ 5 µm). However, considering the high dynamism of the TF, it is unlikely that its thickness is constantly less than 5 µm during OCT scanning, since it is more probably a variable parameter. Accordingly, Napoli and co-workers demonstrated that a non-quiescent model of the TF during visual fixation is more realistic than a perfectly immobile structure [31]. In particular, a wave model of the tear film may explain the cyclic stimulation of photoreceptors (indispensable for the maintenance of vision), the potential activation of specific antioncogenes (for the prevention of tumor diseases), and a continuous degree of dissipation and attenuation of the UV rays for the underlying ocular tissues [32,33,34]. In fact, during visual fixation, despite that the precorneal TF is commonly thought “quiescent” in healthy patients, fixational eye movements occur normally and can transfer energy to human tears (by a continuous motion or vibration), implying a relationship between the nervous system and the ocular surface [35]. Moreover, the bull’s eye pattern of human tears (i.e., the interference pattern in OCT imaging induced by lacrimal movements when eyes are fixating a target) correlated with the heart pulse, thus revealing a previously poorly understood link between the cardiovascular system and the lacrimal functional unit [31].

Nevertheless, precise and direct measurements of the natural TF thickness over time are difficult, not only because of its dynamic nature, but also since the resolution and acquisition speed of commercially available OCT devices are still inadequate for this purpose (i.e., for a quantitative approach) [36]. This aspect is confirmed by the discordant results among various studies. Indeed, in the last twenty years, several authors have attempted to quantify the thickness of the tear film, on the basis that it may represent a valid metric to manage lacrimal gland dysfunction.

The first indirect measurements of TF thickness, carried out before and after the use of contact lens or artificial tears, reported values of 3.3 ± 1.5 μm or 3.3 ± 2.6 μm [37,38]. More recently, using a SD-OCT with one micrometer axial resolution (ultra-high-resolution OCT system) to perform direct measurements, Yadav and co-workers have found values of approximately 5 microns [39]. This value seems to be robust enough, having been reproduced by other technologies, i.e., the Ti: Sapphire laser OCT system and interferometric methods [40,41].

In several pathological or clinical situations, such as in dry eye patients (due to the paucity of tears) or between blinks (due to the rapid fluctuations in thickness values during tear distribution), the minute modifications in TF thickness may be hardly monitored. To minimize this drawback, in order to obtain reliable measurements it is recommended to perform OCT scanning after the physiological spreading of the TF over the whole ocular surface (occurring in approximately 0.5 seconds) [31,32].

The precorneal TF can also be evaluated by OCT imaging to analyze, in real time and non-invasively, the dynamic changes induced by different artificial tears (ATs). We found that the TF morphological pattern is related to the chemical composition of tear substitutes [42]. We observed a raise in OCT reflectivity when lipid concentration is augmented, and an increase in thickness with the use of water-soluble polymers. Overall, three main types of OCT signal were identified in this way, namely the linear, the double-band, and the granular TF pattern. When recording the intensity of the OCT signal from the TF, it is very important to adjust the axial distance of patients so that the ocular surface is within the middle third of the OCT scan [43].

## 5. Tear Meniscus

The tear menisci are compartments of the lacrimal system that can supply tear fluid to the precorneal TF. They function as a lacrimal reservoir since they contain most of the tear volume [44,45]. In physiological conditions, the menisci assume a concave appearance because the tear molecules close to the epithelia are attracted by adhesive forces to the ocular surface, so that surface tension distorts the free surface according to the Young–Laplace equation [46]. Otherwise, if the balance between the cohesive and adhesive forces were different, a horizontal or convex free surface would be observed.

**Figure 5:**
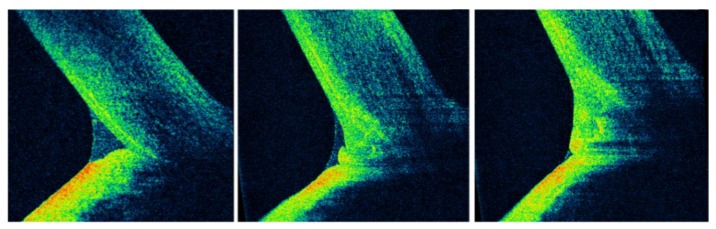
Optical coherence tomography (OCT) imaging of the lower tear meniscus (LTM), lid-parallel conjunctival folds (LIPCOFs), and conjunctivochalasis. The qualitative and quantitative features of the LTM are clearly visible in cross section by means of OCT (left). However, this approach may be invalidated by the presence of LIPCOFs (middle) or conjunctivochalasis (right) [42].

Considering the various difficulties in the direct quantification of the preocular TF thickness, many authors have suggested and employed tear meniscus as an indirect indicator of the overall tear volume and TF dynamics. Indeed, the upper and lower menisci are easier to visualize with most of the commercially available OCT devices (Figure 5).

However, although OCT imaging is capable of measuring quantitatively the tear meniscus in terms of height, depth, radius of curvature and area, none of these variables are predictive of the TF thickness [45]. A relationship of dependence is noted solely between the height of the lower meniscus and the volume of the mucoaqueous layer [47].

The reliability of OCT meniscometry seems to be technology dependent, since SD-OCT systems appear to provide better results in terms of repeatability than TD platforms [48]. This implies that an adequate OCT resolution may avoid measurement biases due to anatomical abnormalities, such as lax conjunctival folds or incongruities in lid apposition (Figure 5) [42,49,50]. However, image analysis is mainly subjective and time-consuming, and thus issusceptible to operator-dependent errors [51]. Some authors have suggested that artifacts in metric parameter measurements may arise immediately after each blink, which implies that OCT scans should be performed at least after 2 seconds [42].

The changes of tear meniscus over time have been measured by several groups during blinks or after instillation of tear substitutes [38,47,52,53]. Some metric variables of the menisci appeared to be reduced in the elderly and in patients with dry eye or thyroid-associated ophthalmopathy. Conversely, higher tear volume was found in MGD patients with corneal damage [47,54,55,56].

After instillation of ATs, we have found that a specific appearance of tear meniscus and an increase in tear volume is related to the type of tear substitute. In particular, it was possible to detect a constellation pattern (with multiple hyper-reflective points), an enhanced reflectivity type (with areas of high/moderate reflectivity), and a hypo-echoic type (characterized by areas of low or no reflectivity) [42].

Although the aforementioned limits need to be taken into account, SD-OCT meniscometry may be considered today a rapid and non-invasive technique to evaluate, in real time, the behavior of human tears.

## 6. Ocular Surface Epithelia

An alternative approach to investigate the lacrimal functional unit is represented by OCT imaging of the anatomical structures of the ocular surface. These mainly include the epithelia of the cornea and conjunctiva (e.g., with its folds and tumors), as well as the meibomian glands or other constituent parts.

### 6.1. Cornea

**Figure 6:**
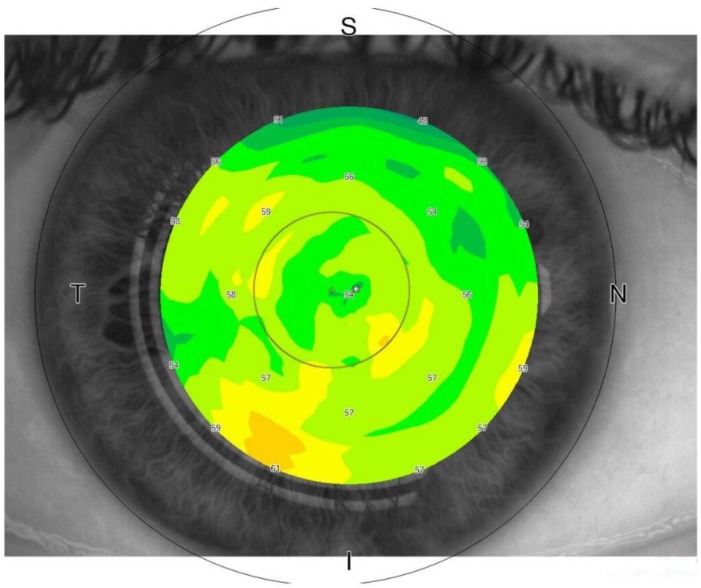
Swept-source (SS) optical coherence tomography (OCT) imaging of the corneal epithelium thickness. The thickness of the corneal epithelium can be accurately quantified by OCT imaging at the central vertex and in the peripheral zones. (Sectors were as follows: N: nasal; T: temporal; S: superior; I: inferior). Reflectivity of the en-face OCT image indicates the corneal epithelial thickness (low intensity (dark blue) ≤ 25 μm to high intensity (red/white) ≥ 85 μm).

**Figure 7:**
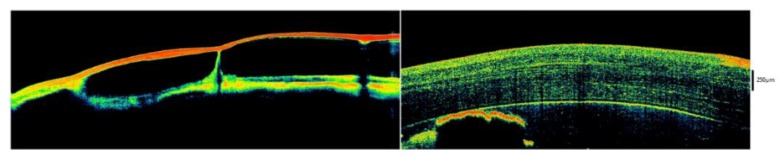
Degeneration of the ocular surface epithelia by portable optical coherence tomography (OCT). Using a portable spectral-domain (SD) OCT in research setting, a progressive degeneration of the ocular surface epithelia and underlying layers can be monitored over time in absence of a physiological tear production. The structural disorganization of the ocular surface epithelia (left), or the loss of corneal epithelium (right), is noninvasively revealed by OCT imaging [59,60,61]. Reflectivity of the en-face OCT image [low intensity = dark/blue to high intensity = red].

The corneal epithelium is a stratified multicellular layer (~50 μm in thickness at the corneal vertex) that performs important defense functions for the eye (Figure 6) [57,58]. Unlike the conjunctiva that is a highly reactive tissue, the cornea is a relatively non-reactive structure since inflammatory processes may alter its transparency [18]. Most of the defense mechanisms of the cornea (e.g., the presence of secretory immunoglobulin A, epithelial electric charge, lysozyme and lactoferrin) are provided by the tear film and secondarily by the limbal vessels. The tear film plays also a fundamental role in oxygenation of the cornea, especially at the central level, where its transparent architecture is devoid of vessels. If tear flow decreases, the corneal epithelium and the underlying layers progressively degenerate over time (Figure 7), as was shown in an experimental work with a portable OCT (which proved to be a reliable tool for this type of evaluation) [59,60,61].

The external surface of the corneal epithelium shows a roughness of ~ 0.5 µm along its contour [62]. It might have a role in the distribution and appearance of the precorneal TF (e.g., a potential relationship with the aforementioned wave pattern of human tears).

Currently, OCT imaging is capable of identifying the anatomical structure and measuring the thickness of various corneal layers, and also of detecting some ultrastructural features (such as the stromal striae departing from the anterior stroma to the Descemet membrane) [7,63,64]. This approach can be useful in keratoconus eyes (where an increased epithelial thickness may be predictive of corneal hydrops) [65], in corneal dystrophies involving the epithelium and/or basal membrane [66], in differential diagnosis between pterygium and pseudo-pterygium [67], after photorefractive keratectomy [68] or cross-linking (during the remodeling of epithelium, and the detection of the demarcation line) [69], and in monitoring the epithelia changes following chemical burns [70].

### 6.2. Conjunctiva

**Figure 8:**
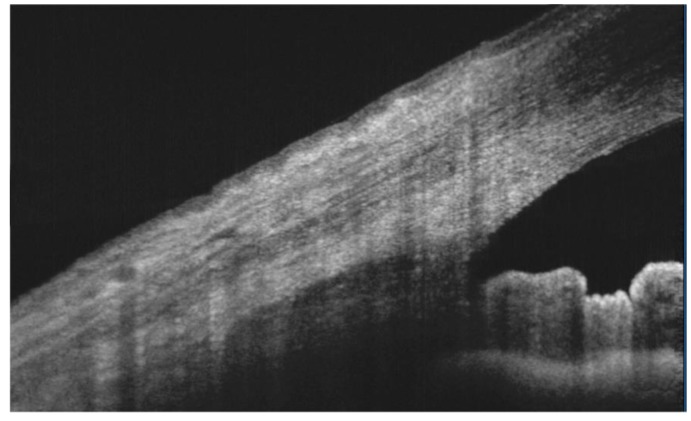
Optical coherence tomography (OCT) imaging of the ocular surface epithelia. The epithelia of the ocular surface (especially conjunctiva, limbus, and cornea) can be accurately visualized and quantified by OCT imaging (as a continuous, hypo-reflective structure). This approach may be useful to diagnose and monitor a wide variety of pathological situations (see text).

The conjunctival epithelium is also a stratified multicellular layer (~45–70 µm in thickness), where cells are connected by adherens junctions (to resist against shear stress) and by tight junctions (to provide a barrier against the external environment) (Figure 8) [57].

Several ocular surface diseases are associated with abnormalities of the conjunctival and corneal epithelia. Among the conjunctival alterations studied by OCT, we mention the lid-parallel conjunctival folds (LIPCOFs) (Figure 5) [71]. LIPCOFs may represent the initial stage of conjunctivochalasis, since they are smaller and appear only laterally, regardless of age [72]. Since LIPCOFs are associated with dry eye disease, some authors have used OCT to assess their structure. A research group reported that excision of conjunctivochalasis may result in an improvement of the TF stability, corneal surface regularity, and contrast sensitivity, as well as the restoration of the tear meniscus [73].

Another application of OCT imaging is represented by the evaluation of ocular surface tumors and their treatment. Several studies demonstrated that OCT may unveil a thickened, hyper-reflective epithelium (~390 µm), which switches abruptly from normal to abnormal appearance (strongly hyper-reflective), in ocular surface neoplasia [67,74,75,76]. Our group investigated the potential of OCT in detecting the changes inthe ocular surface after removal of pinguecula with an argon laser [77].

Moreover, changes in conjunctival epithelium and stroma can also be studied after filtering surgery for glaucoma. In this condition, a quantitative and qualitative analysis of OCT signal is of great importance to evaluate the filtering blebs [43].

### 6.3. Meibomian Glands

The meibomian glands represent a holocrine type of exocrine glands, situated inside the tarsal plates, which secrete an oily substance (i.e., the meibum) at the rim of the eyelids [78]. During blinking, the latter constitutes the outer lipid layer of the tear film that improves the stability of the tear film leading to the formation of a smooth surface at the interface between tear film and air [79,80,81]. This physical quality is essential for an excellent light refraction of the ocular optical system. In case of lipid dysfunctions, ocular surface diseases and ocular discomfort symptoms may occur [78].

**Figure 9:**
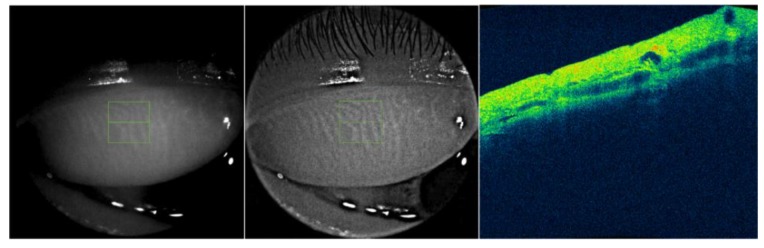
Fourier-domain (FD) optical coherence tomography (OCT) imaging of meibomian glands. Commercial FD-OCTs and custom-built systems can reveal the architecture of meibomian glands and their internal structure. A simple technique to obtain an infrared (IR) meibography by OCT is based on the modulation of image contrast and brightness until an adequate enhancement is achieved. An unmodified IR OCT image of the everted upper lid is displayed (left). After image processing (middle), the same enhanced image can disclose the glandular tissue in detail. Moreover, in cross-sectional OCT scans, it is also possible to detect the internal features of meibomian glands (right) [87]. Black-and-white pictures before (left) [default values of contrast and brightness = 1]) and after (right) [percentage increase in contrast = 140%, percentage decrease in brightness = 6%] image manipulation. Reflectivity of the en-face OCT image (right) [low intensity = dark/blue to high intensity = red].

Traditionally, the structural details of the glands have been evaluated by biopsy or contact meibography, the former being clearly a drastically more invasive technique than the latter [82]. In recent years, OCT imaging of glandular tissue has been shown to be effective, leading to the growing diffusion of this contactless, in vivo, direct, reliable and patient-friendly technique [83,84,85,86]. Several authors have confirmed that meibomian glands can be optimally visualized using commercial OCTs and custom-built systems. Moreover, a good agreement between OCT meibography and traditional lid transillumination has been demonstrated by our group (Figure 9) [87].

Lastly, since today ophthalmologists use OCT devices routinely to image retinal structures, OCT meibography can be easily performed on a standalone basis.

### 6.4. Other Structures of the Ocular Surface

As reported by some authors, OCT imaging is capable of detecting several anatomical elements of the lacrimal gland (ducts, lobules, parenchyma and acini) [88]. However, although this direct and in vivo approach is very interesting, its diagnostic value has not yet been validated.

**Figure 10:**
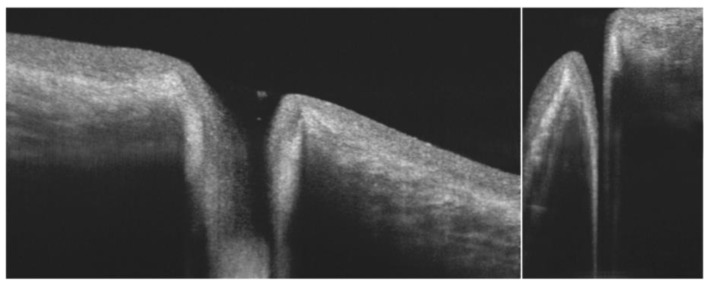
Lacrimal punctal region by optical coherence tomography (OCT). The anatomy of vertical canaliculus and lacrimal punctal region can be directly imaged by commercial OCT systems [89,90].

Similarly, the lacrimal punctal region and the anatomy of vertical canaliculus have been directly imaged and analyzed by commercially available OCT systems and a positive correlation has been found between tear meniscus height and size of the lacrimal punctum (Figure 10) [89,90]. Moreover, corneo-scleral tissue morphology can also be investigated by detecting the Vogt palisades, the blood and lymph vasculature [91,92].

## 7. Tear Film Dynamics

Human tears lubricate, cleanse and nourish the epithelia cells and provide immune and physical protection against environmental harmful agents [1,18]. The tear film represents the most dynamic structure of the lacrimal functional unit: it must react promptly to changes occurring in environmental and/or bodily conditions. For instance, variations in air currents or humidity, or in the bacteriological profile of ocular surface flora, as well as the presence of foreign bodies, are all conditions that may lead to the loss of tear homeostasis and hence require immediate adaptive changes of the system [19].

The dynamics of the lacrimal system imply an adequate production of tears, a uniform spreading and a stable adhesion of tears to the epithelia, as well as their removal (via the nasolacrimal drainage system, the evaporation, and the residual absorption in the surrounding tissues), thus resulting in a continuous and physiological tear turnover [93]. Accordingly, the latter is considered a global index of the integrity of lacrimal system, since it is related to the final result of all these dynamic processes [93,94]. Indeed, a reduced tear clearance was correlated with ocular inflammation, ocular surface diseases, loss of sensitivity and aging [95,96,97].

The dynamic changes of the tear film involve simultaneously and mutually all its three compartments (i.e., preocular, meniscal, and fornical portion), implying radial and tangential movements across the ocular surface [40,98]. However, although the tear turnover should ideally be evaluated in the various compartments, most studies focused on the lower tear meniscus due to its preferable position and size.

In the past, tear turnover has been clinically estimated on the basis of the decay over time of a dye, mainly sodium fluorescein, placed in the tear fluid. However, this traditional approach does not allow for a detailed, non-invasive visualization of tears in cross section, while OCT imaging can provide a series of transversal images allowing a quantitative and qualitative analysis. Moreover, fluorescein sodium destabilizes the tear film, penetrates through the cornea, and may create staining and pseudo-staining of the ocular surface [95,99].

Several attempts have been made to explore the dynamics of the tear film in a direct or indirect way (e.g., after saline instillation) by continuous measurements over time using OCT imaging [53,100,101].

Early phase tear clearance was evaluated by Zheng et al. by monitoring the tear meniscus height and area over time [53]. Although this method represents an interesting technique to measure the tear volume modifications following an increment of 5 µL of saline instillation, the limit of this approach is that tear variations are detectable until the return to baseline metric values, despite the “stagnation” of tears or substances that further persist in the lacrimal meniscus [100]. In addition, the meniscus metrics represent a set of indicators to mainly evaluate the aqueous layer [101].

**Figure 11:**
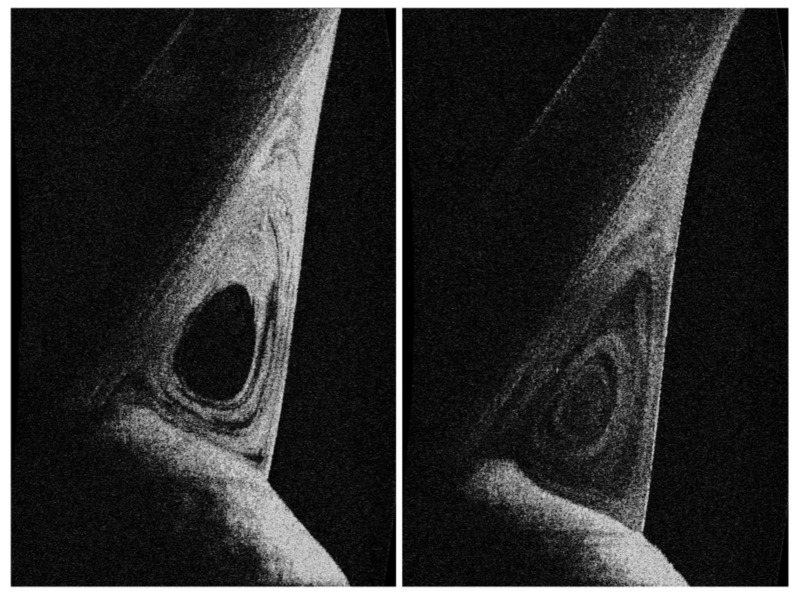
Clearance of human tears in contrast-enhanced optical coherence tomography (OCT) imaging. The flow of lipids in the lower tear meniscus can be reliably observed by OCT imaging after administration of a lipid-based tracer. The latter represents a contrast agent to improve the detection of tear film dynamics. Of note, a vortex pattern in tear distribution may be disclosed in the inter-blink period. OCT imaging was performed in the same conditions of temperature (15 °C–25 °C) and humidity (30%–50%) in a dimly light room [102].

Napoli and co-workers have demonstrated the feasibility of applying a lipid-based tracer (castor oil) to OCT imaging to evaluate the clearance of human tears (in particular, the lipid tear turnover) (Figure 11) [102]. This chemical represents the first example of an OCT contrast agent having the quality to enhance the visualization of tear film dynamics. In this study, it was possible to track the flow of lipids in the lower tear meniscus and to recognize two main distribution patterns, i.e., the vortex type (in which a central area of poor reflectivity is surrounded by concentric areas of elevated reflectivity) and the homogeneous type (in which the meniscus reveals a similar reflectivity in each point, easily classified according to a reflectivity grading scale). Thus, the existence of centrifugal forces in the tear meniscus was demonstrated on the transverse plane. This implies that the “excretion of tear lipids” toward the lid skin occurs not only under conditions of overflow due to a large volume, but also because of a mechanism related to high fluid dynamics characteristics (turbulence of the menisci). In addition, a significant correlation between the tear turnover of lipids and the aqueous flow was found in healthy participants and in patients with dry eye or MGD, thus suggesting that some lipids regularly interact with some proteins (e.g., lipocalin) in the aqueous phase.

Inregards to the preocular TF changes, as mentioned above, the inherent variability in the thickness values of a wave TF is likely to be a significant confounding factor in any type of measurement. Probably, its exact structural feature (thickness at a given point and time) is at present impossible to be determined [18]. Therefore, the behavior of the preocular TF should necessarily be evaluated differently, e.g., with a marker, and taking into account its oscillatory pattern [27,30,31].

Prior to these insights, ultra-high OCT resolution has been used to detect the tear film changes with a series of cross-sectional images and to study the dynamics of the TF thickness (showing a thickness change from 5 µm to 2.5 µm over 15 s time, in healthy subjects). However, further studies are needed to assess the reliability of this approach [41].

## 8. Integrative Model of Tear Film Behavior during the Inter-Blink Period

**Figure 12:**
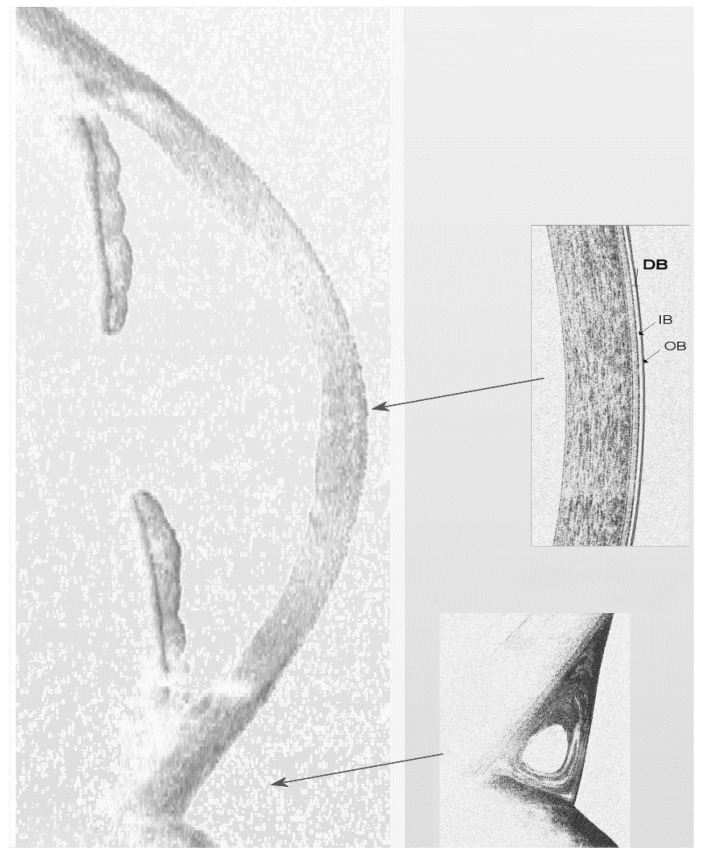
The integrative model of tear film behavior during the inter-blink period and visual fixation. In cross section, it is possible to detect, by optical coherence tomography (OCT), the behavior of the precorneal tear film (TF) as modification over time of the double-band (DB) structure. The latter consists of an inner (IB) and an outer (OB) layer of different reflectivity (small arrows). In addition, the high fluid dynamics of the tear meniscus evidenced by the presence of the vortex distribution pattern (after using a lipid-based tracer that enhances the reflectivity of tears) can also be seen. The continuous dynamism of the TF is the starting point of the model. Within the various areas of the ocular surface, the tear turnover should not be believed as a constant and single parameter since it has proved to be different, for instance, in the precorneal region (large arrow at the top) or in the menisci (large arrow at the bottom). Likewise, the various compounds (e.g., lipids, aqueous, or mucins) also revealed a different washout time. Intriguingly, as demonstrated by OCT imaging, there exists an inverse relationship between the tear clearance above the precorneal surface and that of the menisci, which may dynamically change (even reverse) as the environmental and bodily conditions evolve (e.g., dry eyes exhibit “opposite” behavior with respect to healthy eyes). Moreover, although the precorneal TF in healthy subjects shows an absent or very low clearance during visual fixation, it finely vibrates due to small eye movements, thus supporting a non-quiescent model of human tears.

Much is known today about the complex tear dynamics during the inter-blink interval and visual fixation. In this review we propose an integrative model that synthesizes past research and specifies the relationship between the TF behavior of the precorneal compartment and that of the meniscus. Our depiction is shown in Figure 12.

In healthy subjects, the TF shows excellent adhesive properties on the corneal epithelium (interpretable as an “elevated contact time” or “poor clearance” at the level of the central cornea) and a substantial tear clearance in the menisci [30,31,102]. A prolonged contact time between tears and the corneal surface is useful to promote exchange of metabolites and oxygen by diffusion, and an adequate turnover of the meniscus guarantees a continuous supply of nutrients and the clearing of waste substances.

On the contrary, in dry eyes, an “inflamed precorneal TF” reveals a reduced adhesiveness of the central cornea, thus limiting the contact time of the pro-inflammatory or toxic factors with the epithelium, preserving in this way the corneal transparency and the barrier integrity. In addition, the delayed turnover of tear menisci presumably maintains an appropriate tear volumeso that the frictional forces between the ocular surface and eyelids cannot increase indefinitely.

Interestingly, during visual fixation, an oscillatory model of the precorneal TF has been proven to be more realistic than a perfectly quiescent model [31]. Accordingly, in dry eyes, the paucity of tears (due to poor production or poor stability) implies scarce or absent vibrations on the outermost surface of the tear film. This may explain the blurred vision frequently experienced by dry eye patients. 

Overall, three main features of such a model deserve attention. Firstly, it can be deduced that the tear turnover should not be considered as a single and constant parameter, since it may differ in its chemical composition (e.g., lipids, aqueous, mucins) and its distribution within the various areas of the ocular surface (e.g., precorneal or meniscus). Indeed, although there is a correlation between the various phases (or thermodynamic systems), the time required for the tear turnover of the lipids and the clearance of the aqueous layer is considerably different.

Secondly, an inverse relationship between the tear turnover of the precorneal surface and that of the menisci was demonstrated by different OCT studies. Intriguingly, this finding improved our understanding of tear physiology, such as the existence of specific dynamic functions of the various compartments (or regions of space) of the tear film detectable with OCT.

Thirdly, the model implies that the TF behavior may dynamically change according to environmental and bodily condition modifications (e.g., ocular surface inflammation). Of note, the TF behavior in the precorneal compartment and in menisci can be considered as “opposite” between healthy (where the contact timeis elevated above the cornea and reduced in the menisci) and dry eyes (where the contact timeis decreasedabove the cornea and prolonged in the menisci).

The pivotal point of this model is represented by the continuous dynamism of the TF structure, even when it could be considered as “stable”. Under ideal conditions, the precorneal TF shows a very low or absent clearance (or turnover) during visual fixation but, despite this, it still moves or vibrates due to the small eye movements. The observational evidence of such TF vibration considerably supports a non-quiescentmodel of TF (i.e., a TF in continuous movement), and it may clearly have a variable number of implications, both biological and optical.

## 9. Conclusions and Future Directions

In recent years, OCT imaging has become a more and more valuable tool in the clinical and experimental assessment of the ocular surface. Its main advantages include speed, histological resolution, non-invasiveness, and the possibility to obtain in vivo imaging of the tear film and epithelia. Reliable quantitative measurements of various structures are also possible in real time.

Moreover, OCT imaging from a cross-sectional point of view permits us to explore new aspects of the tear film dynamics across the various compartments of the ocular surface. Accordingly, an integrated model on the relationship between the tear film behavior in the precorneal compartment and that in the meniscusis proposed.

External factors to the model that must be considered concern the OCT technology. More advanced OCT technology is needed to achieve a more detailed detection of TF features. For instance, since axial resolution depends on the light sources, a wider light bandwidth should be evaluated. On the other hand, a better lateral resolution may be obtained by using an alternative focusing lens regarding image depth, or by micro-OCT.

In the near future, artificial intelligence may be used to assist ophthalmologists in the classification of OCT images and in the diagnosis of abnormalities of the lacrimal functional unit, as was the case for some retinal, corneal and neurodegenerative diseases [103,104,105].

In conclusion, future advances in OCT technology may considerably improve our knowledge regarding the dynamics and the complex structure of the lacrimal functional unit.
